# Supplementary material for: Timed topical dexamethasone eye drops improve mitochondrial function to prevent severe retinopathy of prematurity
Source: Angiogenesis. 2024 Sep 17;27(4):903–17. doi: 10.1007/s10456-024-09948-2 (PMC11564262; doi:10.1007/s10456-024-09948-2)
Supplement: Supplementary file 1 — Supplementary Material 1 [file 10456_2024_9948_MOESM1_ESM.docx]

**Timed topical dexamethasone eye drops improve mitochondrial function to prevent severe retinopathy of prematurity.**

Hitomi Yagi^1,2^, Myriam Boeck^1,3^, Mariya Petrishka-Lozenska^4^, Pia Lundgren^4^, Taku Kasai^5^, Gael Cagnone^6,7^, Katherine Neilsen^1^, Chaomei Wang^1^, Jeff Lee^1^, Yohei Tomita^1,2^, Sasha A. Singh^5^, Jean-Sébastien Joyal^6,7,8^, Masanori Aikawa^5,9,10^, Kazuno Negishi^2^, Zhongjie Fu^1^, Ann Hellström^4*^, Lois E.H. Smith^1*^

*Corresponding authors:

Lois EH Smith, MD, PhD (email: lois.smith@childrens.harvard.edu), Department of Ophthalmology, Boston Children’s Hospital, Harvard Medical School, 3 Blackfan Circle, CLS 18, Boston, MA 02115, USA. Tel: (+1) 617 919 2529.

Ann Hellström (email: ann.hellstrom@medfak.gu.se), The Sahlgrenska Centre for Pediatric Ophthalmology Research, Department of Clinical Neuroscience, Institute of Neuroscience and Physiology, Sahlgrenska Academy, University of Gothenburg, Gothenburg, Sweden. Tel:(+46) 31 3434720.

Affiliations:

1. Department of Ophthalmology, Boston Children’s Hospital, Harvard Medical School, Boston, MA 02115, USA
2. Department of Ophthalmology, Keio University School of Medicine, Tokyo, 160-8582, Japan
3. Eye Center, Medical Center, Faculty of Medicine, University of Freiburg, 79106, Freiburg, Germany
4. The Sahlgrenska Centre for Pediatric Ophthalmology Research, Department of Clinical Neuroscience, Institute of Neuroscience and Physiology, Sahlgrenska Academy, University of Gothenburg, Gothenburg, 40530, Sweden
5. Center for Interdisciplinary Cardiovascular Sciences, Division of Cardiovascular Medicine, Department of Medicine, Brigham Women's Hospital, Harvard Medical School, Boston, MA 02115, USA.
6. CHU Sainte-Justine Research Center, Montreal, QC, CA, H3T 1C5
7. Department of Pediatrics, Ophthalmology, and Pharmacology, CHU Sainte-Justine, Université de Montréal, Montreal, QC, CA, H3T 1C5
8. Department of Ophthalmology, Université de Montréal, Montreal, QC, CA, H3T 1J4
9. Center for Excellence in Vascular Biology, Division of Cardiovascular Medicine, Brigham and Women’s Hospital, Harvard Medical School, Boston, MA 02115, USA.
10. Channing Division of Network Medicine, Department of Medicine, Brigham Women's Hospital, Harvard Medical School, Boston, MA 02115, USA.

**SUPPLEMENTARY FIGURES AND LEGENDS**

**SUPPLEMENTARY FIGURES**


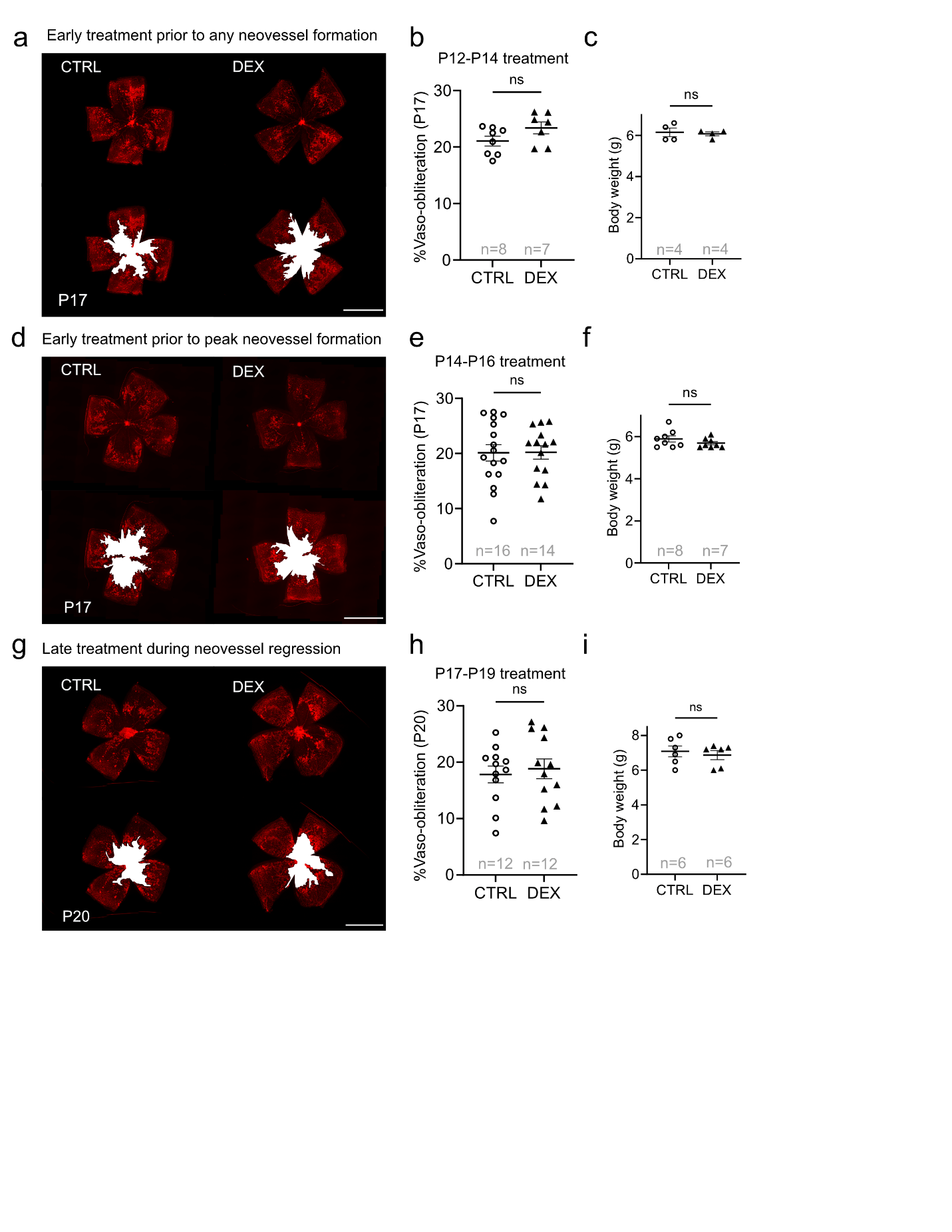


**Supplemental Fig. 1** Dexamethasone eye drops did not alter vaso-obliteration and body weight after daily treatment during OIR.

(**a-i**) OIR retinas treated topically from P12 to P14 (a-c), or P14-P16 (d-f), or P17-P19 (g-i) with one drop per day of dexamethasone eye drops (DEX, 0.1%) or control eye drops (CTRL) with evaluation at P17 (a-f) or P20 (g-i). Representative retinal whole mount images were shown (a, d, g). Retinal vessels were visualized with lectin (red). Vaso-obliterated areas (VO) was highlighted in white. Scale bar, 2mm.The percentage of VO area over the area of retinal whole mount was calculated (b, e, h) and body weight of mice were also shown (c, f, i). n number of each group was labeled in the graphs (each dot represents the number of retinas for VO, or the number of mice for body weight). Two-tailed unpaired t-test; ns, not significant. Mean values ± SEM.


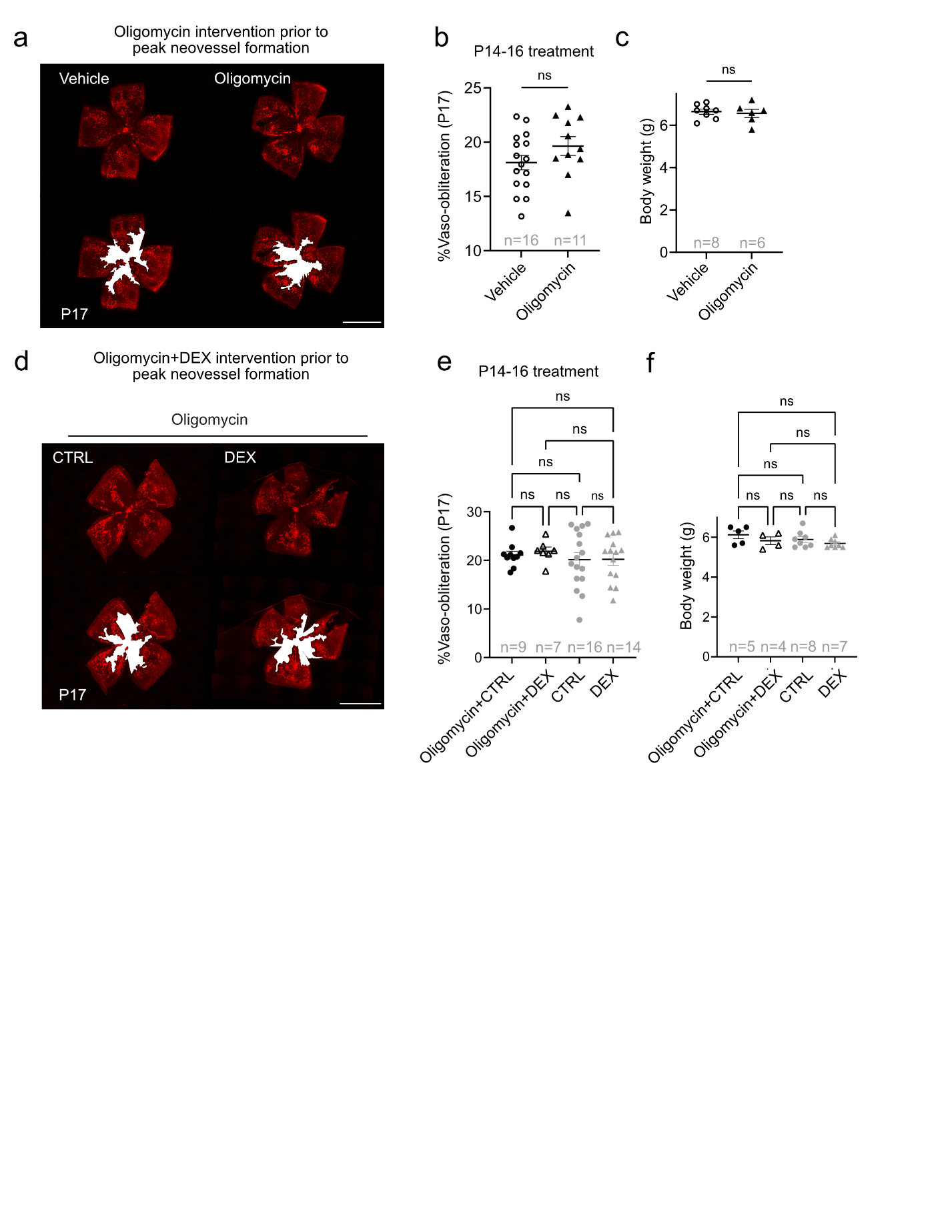


**Supplemental Fig. 2** Oligomycin intervention with or without 0.1% dexamethasone eye drops prior to peak neovessel formation (P14-16) did not change vaso-obliteration and body weight.

(**a-c**) P17 OIR pups injected with Vehicle or Oligomycin (0.25ug/g) i.p., daily from P14 to P16. Representative retinal whole mount image (a) and with vessels in red (lectin) and VO is highlighted in white. Scale bar, 2mm. The percentage of VO area over the area of total retinal whole mount area (b) and body weight (c) was compared between oligomycin and vehicle group. n number of each group was labeled in the graphs (each dot represents the number of retinas for VO, or the number of mice for body weight). Two-tailed unpaired t-test; ns, not significant. Mean values±SEM. (**d-f**) P17 OIR retinas treated from P14 to P16 with oligomycin (0.25ug/g, i.p. daily) plus daily dexamethasone eye drops (Oligomycin+DEX) or control eye drops (Oligomycin+CTRL). Representative retinal whole mount image (d) with vessels red (lectin) and VO highlighted in white. Scale bar, 2mm. The percentage of VO area over the area of total retinal whole mount (e) and body weight (f) was compared. n number of each group was labeled in the graphs (each dot represents the number of retinas for VO, or the number of mice for body weight). DEX and CTRL groups from supplementary figure 1F are included for comparison. One-way ANOVA; ns, not significant. Mean values ± SEM.


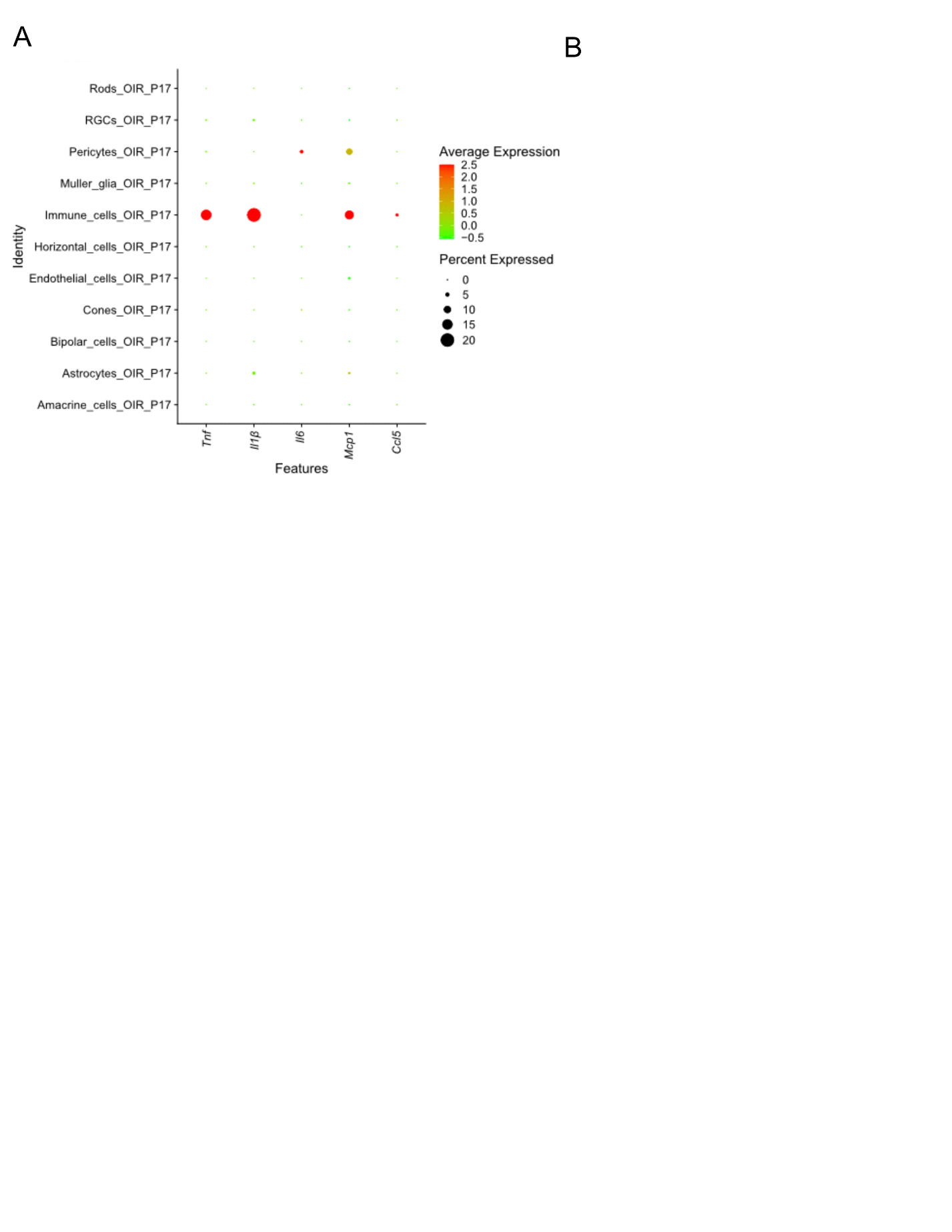


**Supplemental Fig. 3** Pro-inflammatory genes (*Tnf, Il1b, Mcp1, Ccl5*) were predominantly expressed in immune cells in OIR. Gene expression of inflammatory markers with single-cell transcriptomic analysis of P17 OIR mouse retinas (Binet F et al. *Science* 369, (2020)). ‘Percent Expressed’ is the percentage of cells that have one or more transcripts for the gene of interest; ‘Average expression’ is the average transcript count amongst the cells of a cluster. Transcript counts are scaled around zero (zero=the mean across all cells of the dataset).

**Supplementary Table 1:** Primers for real-time quantitative PCR (RT-qPCR)

| *Cox4i2* | CTGCCCGGAGTCTGGTAATG | CAGTCAACGTAGGGGGTCATC |
| --- | --- | --- |
| *Cox6a1* | TCAACGTGTTCCTCAAGTCGC | AGGGTATGGTTACCGTCTCCC |
| *Atp6v1a* | CCGTACTCCGCACTGGTAAAC | TGGGGATGTAGATACTTTGGGT |
| *Apt6v1b2* | ATGCGGGGAATCGTGAACG | AGGCTGGGATAGGTAGTTCCG |
| *Tufm* | GCAGCCACTCTATTGCGAG | CGCGACACAAGAAGGGTGA |
| *Tomm70* | TGGGGCTATGTACCTGTGGAG | GCTTGCTCGTATTTTCCTGCTT |
| *Tnf* | CATCTTCTCAAAATTCGAGTGACAA | TGGGAGTAGACAAGGTACAACCC |
| *Il1b* | TTCAGGCAGGCAGTATCACTC | GAAGGTCCACGGGAAAGACAC |
| *Il6* | TAGTCCTTCCTACCCCAATTTCC | TTGGTCCTTAGCCACTCCTTC |
| *Mcp1* | TAGGCTGGAGAGCTACAAGAGG | AGTGCTTGAGGTGGTTGTGG |
| *Ccl5* | GCTGCTTTGCCTACCTCTCC | TCGAGTGACAAACACGACTGC |
| *Vegfa* | CTGCCGTCCGATTGAGACC | CCCCTCCTTGTACCACTGTC |
| *Vegfr2* | TTTGGCAAATACAACCCTTCAGA | GCTCCAGTATCATTTCCAACCA |
| *Epo* | ACAAAGCCATCAGTGGTCTACG | TCTGGAGGCGACATCAATTCC |
| *EpoR* | TTCCTGCCCGTCTGACTTG | CCAGGATGGTGTACTCAAAGCTG |
| *Cyclophillin A* | CAGACGCCACTGTCGCTTT | TGTCTTTGGAACTTTGTCTGCAA |
